# Carbon-ion radiotherapy alone vs. standard dose photon radiation with carbon-ion radiotherapy boost for high-grade gliomas: a retrospective study

**DOI:** 10.1186/s12885-024-12606-x

**Published:** 2024-07-13

**Authors:** XiaoJun Li, YanShan Zhang, YanCheng Ye, SuQing Tian, TingChao Hu, HongYu Chai, TianE Zhang, Faxin Wen

**Affiliations:** 1 Heavy Ion Radiotherapy Department, Wuwei Cancer Hospital & Institute, Wuwei Academy of Medical Sciences, No. 31 Sanitary Lane, Haizang Road, Wuwei, 733000 Gansu Province China; 2 Department of Radiation Oncology, Peking University Third Hospital, Beijing, 100191 China

**Keywords:** High-grade glioma, Carbon ion radiotherapy, Progression-free survival, Overall survival

## Abstract

**Background:**

This study aimed to compare the survival outcome and side effects in patients with primary high-grade glioma (HGG) who received carbon ion radiotherapy (CIRT) alone or as a boost strategy after photon radiation (photon + CIRT_boost_).

**Patients and methods:**

Thirty-four (34) patients with histologically confirmed HGG and received CIRT alone or Photon + CIRT_boost,_ with concurrent temozolomide between 2020.03–2023.08 in Wuwei Cancer Hospital & Institute, China were retrospectively reviewed. Overall survival (OS), progression-free survival (PFS), and acute and late toxicities were analyzed and compared.

**Results:**

Eight WHO grade 3 and 26 grade 4 patients were included in the analysis. The median PFS in the CIRT alone and Photon + CIRT_boost_ groups were 15 and 19 months respectively for all HGG cases, and 15 and 17.5 months respectively for grade 4 cases. The median OS in the CIRT alone and Photon + CIRT_boost_ groups were 28 and 31 months respectively for all HGG cases, and 21 and 19 months respectively for grade 4 cases. No significant difference in these survival outcomes was observed between the CIRT alone and Photon + CIRT_boost_ groups. Only grade 1 acute toxicities were observed in CIRT alone and Photon + CIRT_boost_ groups. CIRT alone group had a significantly lower ratio of acute toxicities compared to Photon + CIRT_boost_ (3/18 vs. 9/16,* p* = 0.03). No significant difference in late toxicities was observed.

**Conclusion:**

Both CIRT alone and Photon + CIRT_boost_ with concurrent temozolomide are safe, without significant differences in PFS and OS in HGG patients. It is meaningful to explore whether dose escalation of CIRT_boost_ might improve survival outcomes of HGG patients in future randomized trials.

**Supplementary Information:**

The online version contains supplementary material available at 10.1186/s12885-024-12606-x.

## Introduction

High-grade glioma (HGG) is the most commonly diagnosed and aggressive form of brain cancer in adults. The current NCCN guidelines recommend standard treatment of maximal safe resection for resectable lesions, followed by concurrent chemoradiotherapy with temozolomide and 6 cycles of adjuvant temozolomide chemotherapy [12]. For unresectable lesions, biopsy or subtotal resection is performed, followed by concurrent chemoradiotherapy and adjuvant chemotherapy [12]. Despite treatment, the median survival time for patients with glioblastoma, the most aggressive HGG, is around 15–18 months [7, 14, 15, 23].

Carbon ion radiotherapy (CIRT) is a type of heavy particle radiotherapy with distinct physical and biological properties compared to standard photon therapy. It has higher conformity and steeper dose gradients due to the Bragg peak [4]. CIRT also exerts significantly stronger biological effects than conventional X-rays and proton beams, primarily by inducing double-strand DNA breaks. The relative biological effectiveness (RBE) of carbon ions in treating glioblastoma ranges between 3 and 5 [28]. Due to these unique attributes, CIRT might be a promising treatment approach for radioresistant tumors in critical organs, such as gliomas in the brain [18]. CIRT can potentially provide clinical benefits for gliomas [28]. Clinical trials have explored the use of CIRT as an adjunctive boost to conventional photon radiotherapy postoperatively, in combination with concurrent chemotherapy [13, 19, 28], or alone for WHO grade 2 diffuse astrocytomas [11]. In a phase I/II clinical trial conducted in Japan, patients with glioma were treated with photon therapy and chemotherapy followed by CIRT. CIRT dose escalation from 16.8 GyE to 24.8 GyE resulted in a median overall survival (OS) of 35 months for anaplastic astrocytoma patients and 17 months for glioblastoma patients [19]. Another study in China involving 50 patients (34 with glioblastomas and 16 with anaplastic gliomas) treated with proton therapy or a proton plus carbon ion boost reported 12- and 18-month OS rates of 87.8% and 72.8%, respectively [13]. For CIRT alone in patients with WHO grade 2 diffuse astrocytomas, the median progression-free survival (PFS) was 18 months for the low-dose (46.2 GyE) group and 91 months for the high-dose group (55.2 GyE) [11].

According to data from a previous phase I/II trial, CIRT is generally safe for normal brain tissues, providing a foundation for treating gliomas with carbon ion beams [24]. However, Kong et al.’s reported 11 cases of grade I-II late side effects of radiation-induced brain necrosis after particle therapy [13]. Theoretically, carbon ions are high-linear energy transfer (LET) radiation with a high RBE value in the peak region. It causes significant tissue damage due to their high biological effectiveness. In the case of glioma target areas, an area up to 2 cm outside the GTV needs to be irradiated in the subclinical region. However, carbon ions to these regions may cause unpredictable radiation damage to normal brain tissue. Severe late toxicities (necrosis) in the normal brain after irradiation with CIRT alone was observed in one previous study [11]. Therefore, CIRT-induced brain injury cannot be ignored during therapy.

Exploring how to better utilize the physical and dosimetric advantages of carbon ions to improve tumor control, while reducing the incidence of high-dose radiation damage to surrounding brain tissues can provide new treatment recommendations for glioma radiotherapy. The current dose-adverse reaction relationship of carbon ions acting on brain tissue remains unclear. How carbon ions can be safely and rationally used for glioma treatment and the corresponding radiobiological effects of radiation brain injury need to be further studied. In this study, we retrospectively analyzed the survival outcome (OS and PFS) and side effects in patients with primary HGG and received CIRT alone or as a boost strategy after photon radiation in Wuwei Cancer Hospital & Institute, China from 2020.03–2023.02.

## Materials and methods

### Patients reviewed

This study was approved by the Ethical Committee of Wuwei Cancer Hospital & Institute, China (Approval no. 2022-ethicalcheck-16). Thirty-four (34) patients with histologically confirmed high-grade glioma and received CIRT alone or Photon + CIRT_boost_ between 2020.03–2023.08 in Wuwei Cancer Hospital & Institute, China were retrospectively reviewed. Informed consent was obtained from all patients included. The following criteria were applied to select appropriate patients for this analysis:

1. Age ≥ 14 and ≤ 80 years old;
2. High-grade gliomas that can be diagnosed according to the 5th edition of the "WHO Classification of Central Nervous System Tumors" including grade 3 oligodendroglioma (1p/19 codeleted, IDH-mutant); grade 3 IDH-mutant astrocytoma; grade 4 IDH-mutant astrocytoma, grade 4 IDH wild-type glioblastoma and pediatric-type diffuse high-grade gliomas [21].
3. Patients with these tumors, regardless of the completeness of the surgery (total resection, subtotal resection or partial resection after surgery, as well as after stereotactic or open biopsy.
4. No history of other malignant tumors (except for cured skin cancer and stage 0 cervical cancer).
5. Patients received photon radiotherapy (Volumetric Modulated Arc Therapy, VMAT or Intensity-modulated radiation therapy, IMRT, 50 Gy/25F) plus CIRT boost (CIRT_boost_, 24.8 Gy (RBE)/8 Fx) (defined as Photon + CIRT_boost_) or CIRT alone (60.0 Gy (RBE)/16Fx). Concurrent and adjuvant TMZ was used for all patients (Concurrent TMZ 75 mg/m^2^, qd; adjuvant TMZ 150–200 mg/m^2^, D1-5/28 days).

### Radiation strategy

The therapeutic decision of Photon + CIRT_boost_ or CIRT alone was made by full consultation with the patients, with known pros and cons fully communicated. Patients included in this study received the standardized radiation strategy below:

Normal Tissue Dose Constraints: Photon radiotherapy dose constraints for first course: Visual pathway (evaluate optic chiasm and optic nerve separately) D1 < 54 Gy; brainstem D1 ≤ 54 Gy, V60 Gy < 1% PRV; temporal lobe V60 Gy ≤ 1%; spinal cord V50Gy < 1% PRV, Dmax < 45 Gy; eyeball Dmean < 35 Gy; lens Dmean < 6 Gy, D1 < 8 GyE; cochlea V55 Gy < 5%, Dmean < 36 Gy; hippocampus V40 < 5.0 Gy, Dmean < 7 Gy, Dmax < 10 Gy.

CIRT dose constraints for the second course: during the second-course planning and target delineation, distance from the normal organs at risk (OAR) such as visual pathway and brainstem were kept > 3 mm. SOBP region was conformed to GTV as much as possible. Thus normal tissue dose constraints are: Visual pathway (evaluate optic chiasm and optic nerve separately) D20 < 4 Gy (RBE); brainstem Dmax < 5 Gy (RBE), D1 ≤ 3 Gy (RBE); hippocampus V40 < 2.5 Gy (RBE), Dmean < 3 Gy (RBE), Dmax < 7 Gy (RBE).

Target delineation followed guidelines in Diagnosis and Treatment Guidelines for Glioma (2022 Edition) by National Health Commission of China. Target definition was performed according to the NCCN guidelines 2022. Gross tumor volume (GTV) is MRI T1 enhancement or T2-FLAIR abnormal signals and surgical cavity, excluding peritumoral edema. Clinical target volume (CTV)1 expands GTV by 2 cm, for bony structures, ventricles, falx cerebri, tentorium cerebelli, optic apparatus, brainstem etc., expand by 0–0.5 cm, edema is included in CTV1 of first course. CTV2 of second course only includes MRI T1 enhancement or T2-fluid attenuated inversion recovery (FLAIR) abnormal signals, residual/recurrent tumors and/or postoperative cavity with appropriate expansions. planning target volume (PTV) 1 and PTV2 expand CTV1 and CTV2 by 1–3 mm to account for setup errors plus particle range uncertainties.

Prescription doses followed an early publication [19]. PTV1 receives photon radiotherapy first (starting within 30 days post-op), 50 Gy in 25 fractions. After photon RT, PTV2 receives carbon ion RT, 24.8 Gy (RBE) in 8 fractions; 3.1 Gy (RBE)/fx; 1fx/day, 5 days/week. A 95% prescription dose line should cover 95% of CTV or GTV while ensuring normal tissues are tolerable.

Eclipse 15.5 was used for photon planning. ci-Plan was used for passively scattered carbon ion planning. After carbon ion plan was approved, compensators were fabricated. Dose was verified before the plan was transferred to the ciTreat system for treatment.

### TMZ administration

The stupp regimen was used for TMZ administration. Concurrent oral TMZ (75 mg/(m^2^·d)) was provided during radiotherapy for 42 days. 4 weeks after the completion of concurrent chemoradiotherapy, adjuvant chemotherapy stage was initiated. Oral TMZ (150–200 mg/(m^2^·d)) was provided for 5 days, repeated every 28 days, a total of 6 cycles.

### Therapeutic responses and Toxicity assessment

Patients were followed and the therapeutic responses (Complete response (CR), partial response (PR), disease stability (SD), and disease progression (PD)) were assessed following The Response Evaluation Criteria in Solid Tumors (RECIST) [9].

Toxicities that occurred either during or within three months of starting CIRT were categorized as acute toxicities. On the other hand, toxicities that developed after three months from the initiation of CIRT or persisted for at least three months were classified as late toxicities. Both acute and late toxicities were assessed and scored according to the CTCAE, v4.03.

### Statistical analysis

Progression-free survival (PFS) was defined as the time interval from the date of diagnosis to the date of disease progression or recurrence. Overall survival (OS) was defined as the time interval from the date of pathological diagnosis of high-grade glioma (HGG) to the date of death from any cause. Survival difference was assessed by generating Kaplan–Meier curves, with log-rank tests to compare the statistical difference. Characteristic and toxicity comparison between CIRT alone and Photon + CIRT_boost_ was conducted by Fisher's exact or Chi-square test, except age comparison was performed using unpaired t-test. The Cox’s proportional hazards model [6] was applied for univariate analysis of therapeutic strategies (CIRT alone vs. Photon + CIRT_boost_) for PFS and OS. The assumption of proportional hazards was examined by the Schoenfeld residuals and log–log survival plots. *p* < 0.05 was considered statistical significance.

## Results

### Patient characteristics

**Fig. 1:**
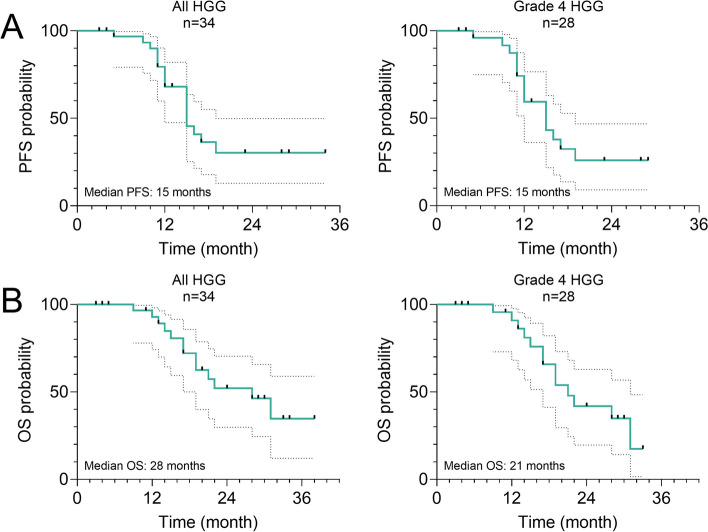
PFS and OS of all cases included in this study. **A**, **B** Kaplan–Meier curves for PFS (A) and OS (**B**) were generated to show the survival of all HGG (left) and grade 4 HGG cases included in this study. Asymmetrical 95%CI was estimated (dot curves). Log-rank p values were calculated

Consecutive and non-selected 34 patients with histologically confirmed high-grade glioma meeting the inclusion criteria described above were retrospectively reviewed. Their clinical characteristics are summarized in Table 1. The 34 cases included 6 WHO grade 3 HGG and 28 WHO grade 4 HGG. Eighteen (18) cases received CIRT alone and 16 had Photon + CIRT_boost_ therapy (Table 1). IDH1/2 gene mutation status was confirmed in 32 cases (Table 1). Then, we compared the clinical characteristics between CIRT alone and Photon + CIRT_boost_ therapy groups. No significant differences were observed in gender, age, WHO grade, KPS scores, IDH1/2 gene mutations, or *MGMT* gene promoter methylation between the two groups (Table 2). The PFS and OS of all HGG and grade 4 cases are presented in Fig. 1. Median PFS was 15 months in both all HGG and grade 4 HGG (Fig. 1A). Median OS was 28 and 21 months in all HGG and grade 4 HGG (Fig. 1B).

**Table 1:** The characteristics of 34 patients included in this study

Characteristics	No. of patients
**Gender**	
Male	14
Female	20
**Age**	
Median (range)	51.1 (16–85)
**Therapeutic group**	
CIRT alone	18
Photon + CIRT_boost_	16
**Pathological diagnosis**	
Grade 3 IDH-mutant astrocytoma	4
Grade 3 oligodendroglioma	2
Grade 4 diffuse midline glioma	2
Grade 4 glioblastoma	26
**KPS score before therapy**	
> 80	13
< = 80	18
NA	3
**IDH1/2 gene**	
Mutant	13
Wild-type	19
NA	2
**MGMT promoter methylation**	
Methylated	13
Unmethylated	5
NA	16

*NA* Not available

**Table 2:** Comparison of the characteristics between CIRT alone and Photon + CIRT_boost_ groups

Characteristics	Therapeutic group		
	**CIRT alone (*N* = 18)**	**Photon + CIRT**_**boost**_** (*n* = 16)**	***p* value**
**Gender**			
Male	7	7	> 0.99
Female	11	9	
**Age**			
Mean ± SD	50.5 ± 18.1	51.8 ± 13.7	0.81
**WHO grade**			
Grade 3	4	2	0.66
Grade 4	14	14	
**KPS score before therapy**			
< = 80	8	10	0.72
> 80	7	6	
NA	3	0	
**IDH gene**			
Mutant	6	7	0.36
Wild-type	10	9	
NA	2	0	
**MGMT promoter methylation**			
Methylated	8	5	0.78
Unmethylated	2	3	
NA	8	8	

Unpaired t-test was conducted for age comparison; Fisher's exact or Chi-square test was applied for other groups

### PFS and OS comparison between CIRT alone and Photon + CIRT_boost_ groups

The median follow-up for the CIRT alone and Photon + CIRT_boost_ groups were 16.00 months (range, 3–38 months) and 13.00 months (range, 3–31 months). 13 patients had died at the time of this analysis (*n* = 9 in CIRT alone group and *n* = 4 in Photon + CIRT_boost_ group).

**Fig. 2:**
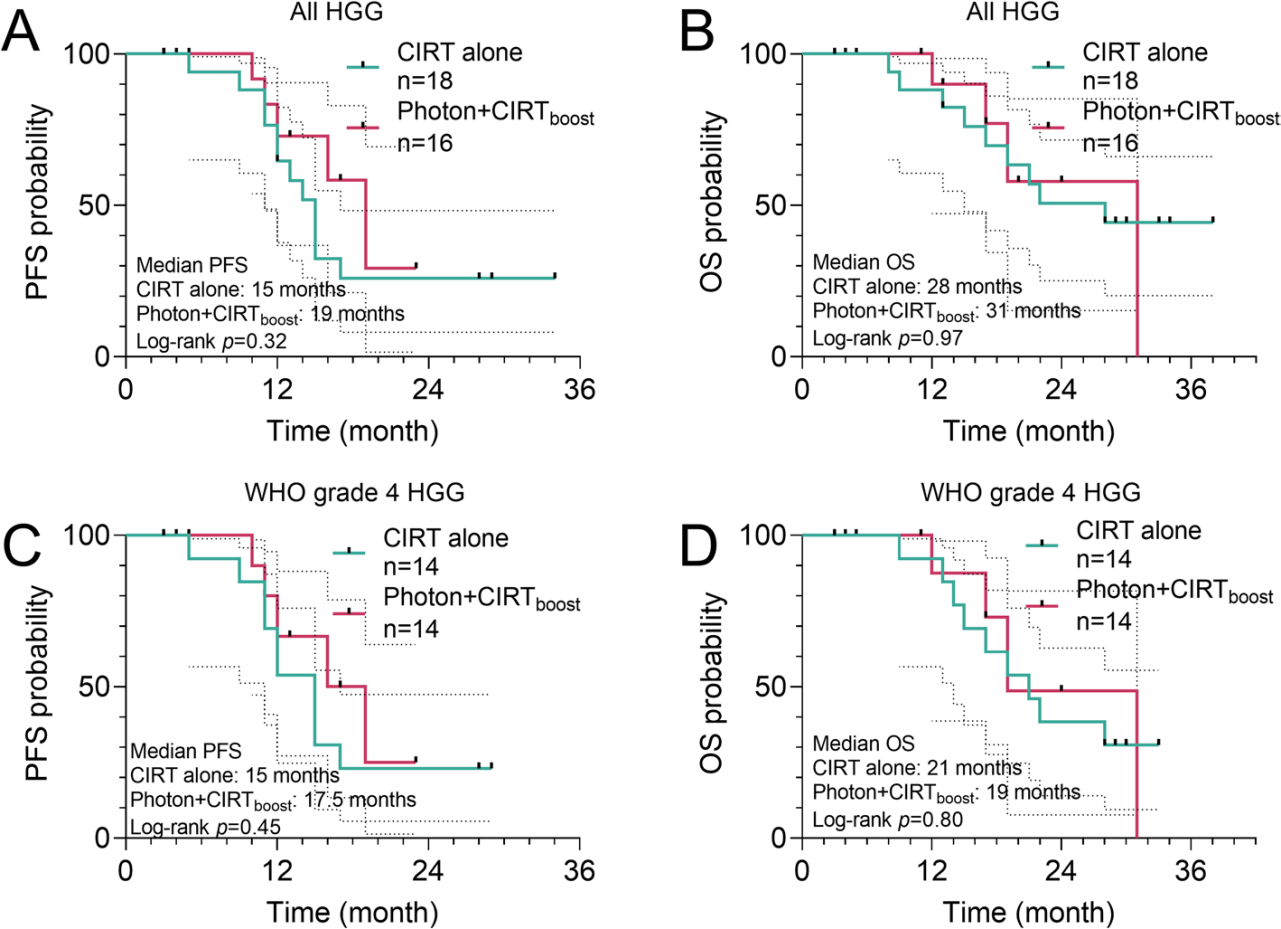
PFS and OS comparison between CIRT alone and Photon + CIRT_boost_ groups. **A-D** Kaplan–Meier curves for PFS (**A** and **C**) and OS (**B** and **D**) were generated to compare the survival differences between CIRT alone and Photon + CIRT_boost_ groups. Asymmetrical 95%CI was estimated (dot curves). Log-rank p values were calculated

For all HGG cases in CIRT alone group, the 12- and 18-month PFS rates were 64.7% (95%CI, 37.7%-82.3%) and 25.88% (95%CI, 8.1%-48.3%), respectively (Fig. 2A). In the Photon + CIRT_boost_ group, the 12- and 18-month PFS rates were 72.92% (95%CI, 36.77%-90.5%) and 58.3% (95%CI, 21.2%-82.9%), respectively (Fig. 2A). The median PFS of the CIRT alone and Photon + CIRT_boost_ group were 15 months and 19 months, respectively. Although the PFS seems longer in the Photon + CIRT_boost_ group than in the CIRT alone group, no significant difference was observed (log-rank *p* = 0.32) (Fig. 2A). In the univariate Cox regression model, the risk of progression was similar between the two therapeutic strategies (CIRT alone vs. Photon + CIRT_boost_, HR: 1.678, 95%CI: 0.589–4.780, *p* = 0.332) (Supplementary Table 1).

For all HGG cases in CIRT alone group, the 12-, 18- and 24-month OS rates were 100%, 63.4% (95%CI, 35.8%-81.6%), and 50.7% (95%CI, 25.1%-71.6%) respectively (Fig. 2B). In Photon + CIRT_boost_ group, the 12-,18- and 24-month OS rates were 90.0% (95%CI, 47.3%-98.5%), 57.9% (95%CI, 15.3%-85.2%) and 57.9% (95%CI, 15.3%-85.2%) respectively (Fig. 2B). The median OS of the CIRT alone and the Photon + CIRT_boost_ group were 28 and 31 months, respectively. No significant difference was observed (log-rank *p* = 0.97) (Fig. 2B). In the univariate Cox regression model, the risk of death was similar between the two therapeutic strategies (CIRT alone vs. Photon + CIRT_boost_, HR: 0.926, 95%CI: 0.278–3.083, *p* = 0.900) (Supplementary Table 2).

For cases with grade 4 HGG in the CIRT alone group, the 12- and 18-month PFS rates were 53.9% (95%CI, 24.8%-76.0%) and 23.1% (95%CI, 5.6%-47.5%), respectively (Fig. 2C). In the Photon + CIRT_boost_ group, the 12- and 18-month PFS rates were 66.7% (95%CI, 27.2%-88.1%) and 50.0% (95%CI, 13.4%-78.7%), respectively (Fig. 2C). The median PFS was 10 and 17.5 months, respectively. Although the PFS seems longer in the Photon + CIRT_boost_ group than in the CIRT alone group, no significant difference was observed (log-rank *p* = 0.45) (Fig. 2C). In the univariate Cox regression model, the risk of progression was similar between the two therapeutic strategies (CIRT alone vs. Photon + CIRT_boost_, HR: 1.546, 95%CI: 0.527–4.535, *p* = 0.427) (Supplementary Table 3).

For cases with grade 4 HGG in the CIRT alone group, the 12-,18- and 24-month OS rates were 100%, 61.5% (95%CI, 30.8%-81.8%) and 38.5% (95%CI, 14.1%-62.8%), respectively (Fig. 2D). In the Photon + CIRT_boost_ group, the 12-, 18- and 24-month OS rates were 87.5% (95%CI, 38.7%-98.1%), 72.9% (95%CI, 27.6%-92.5%) and 48.6% (95%CI, 7.7%-81.6%), respectively (Fig. 2D). The median OS of the CIRT alone and the Photon + CIRT_boost_ group were 21 and 19 months, respectively. No significant difference was observed (log-rank *p* = 0.80) (Fig. 2D). In the univariate Cox regression model, the risk of death was similar between the two therapeutic strategies (CIRT alone vs. Photon + CIRT_boost_, HR: 1.152, 95%CI: 0.347–3.822, *p* = 0.818) (Supplementary Table 4).

### Therapeutic responses

The therapeutic responses of the CIRT alone and the Photon + CIRT_boost_ group were summarized in Table 3. Generally, no significant differences were observed in DCRs or ORRs between CIRT alone and Photon + CIRT_boost_ groups, either in all HGG or WHO grade 4 only cases (Table 3).

**Table 3:** The therapeutic response evaluation

Responses	6 months			12 months			18 months			24 months		
**All HGG**	CIRT alone	Photon + CIRT_boost_	*p* value	CIRT alone	Photon + CIRT_boost_	*p* value	CIRT alone	Photon + CIRT_boost_	*p* value	CIRT alone	Photon + CIRT_boost_	*p* value
CR	1	0		1	1		1	0		1	0	
PD	0	0		5	3		4	1		2	1	
PR	10	9		6	7		0	1		5	1	
SD	6	3		4	1		4	2		0	0	
DCR	100.0%	100.0%	> 0.99	68.8%	75.0%	> 0.99	55.6%	75.0%	> 0.99	75.0%	50.0%	> 0.99
ORR	64.7%	75.0%	0.69	43.8%	66.7%	0.28	11.1%	25.0%	> 0.99	75.0%	50.0%	> 0.99
**WHO Grade 4 only**												
CR	1	0		1	1		1	0		1	0	
PD	0	0		5	3		2	1		1	1	
PR	7	8		3	5		0	1		3	1	
SD	5	2		3	1		3	1		0	0	
DCR	100.0%	100.0%	> 0.99	58.3%	70.0%	0.67	66.7%	66.7%	> 0.99	80.0%	50.0%	> 0.99
ORR	61.5%	80.0%	0.41	33.3%	60.0%	0.39	16.7%	33.3%	> 0.99	80.0%	50.0%	> 0.99

*CR* Complete response, *PR* Partial response, *SD* Stable disease, *PD* Progressive disease, *ORR* Objective response rate, *DCR* Disease control rate

At 12 months, the DCRs were 68.8% and 75.0% for all HGG cases in the CIRT alone and the Photon + CIRT_boost_ group, respectively (Table 3). In WHO grade 4 cases, the DCRs were 58.3% and 70.0% respectively (Table 3). The ORRs were 43.8% and 66.7% in the CIRT alone and the Photon + CIRT_boost_ group for all HGG cases, respectively (Table 3). In WHO grade 4 cases, the ORRs were 33.3% and 60.0%, respectively (Table 3).

### Subgroup analysis by gender, grade, IDH1/2 mutation status, and KPS scores

**Fig. 3:**
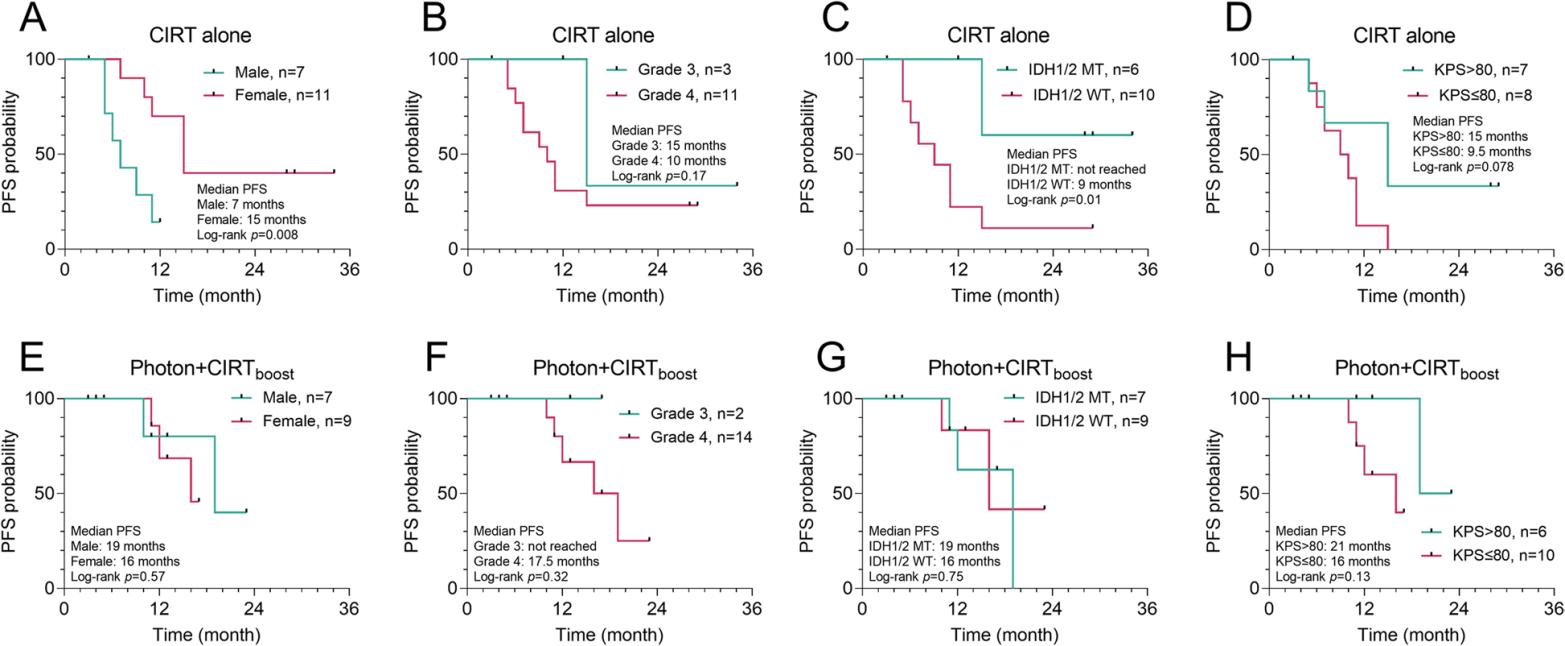
Subgroup analysis of PFS in patients with different clinical characteristics. **A-H** In patients who received CIRT alone (**A**-**D**) or Photon + CIRT_boost_ (**E**–**H**), Kaplan–Meier curves for PFS were generated to compare the survival differences between gender (**A** and **E**), WHO grade 3 and 4 (**B** and **F**), IDH1/2 wild-type (WT) and mutant (MT) (**C** and **G**) and KPS > 80 and ≤ 80 (**D** and **H**)

**Fig. 4:**
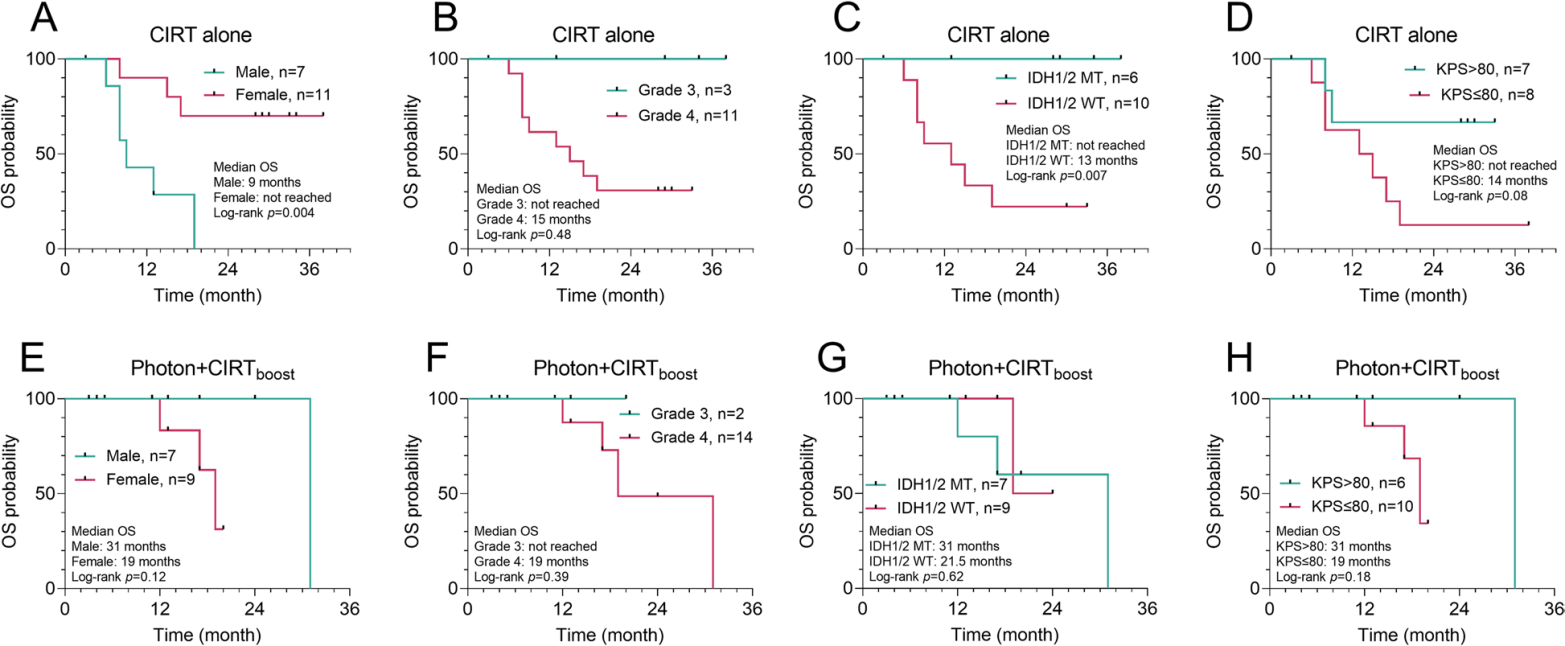
Subgroup analysis of OS in patients with different clinical characteristics. **A-H** In patients who received CIRT alone (**A**-**D**) or Photon + CIRT_boost_ (**E**–**H**), Kaplan–Meier curves for OS were generated to compare the survival differences between gender (**A** and **E**), WHO grade 3 and 4 (**B** and **F**), IDH1/2 wild-type (WT) and mutant (MT) (**C** and **G**) and KPS > 80 and ≤ 80 (**D** and **H**)

**Fig. 5:**
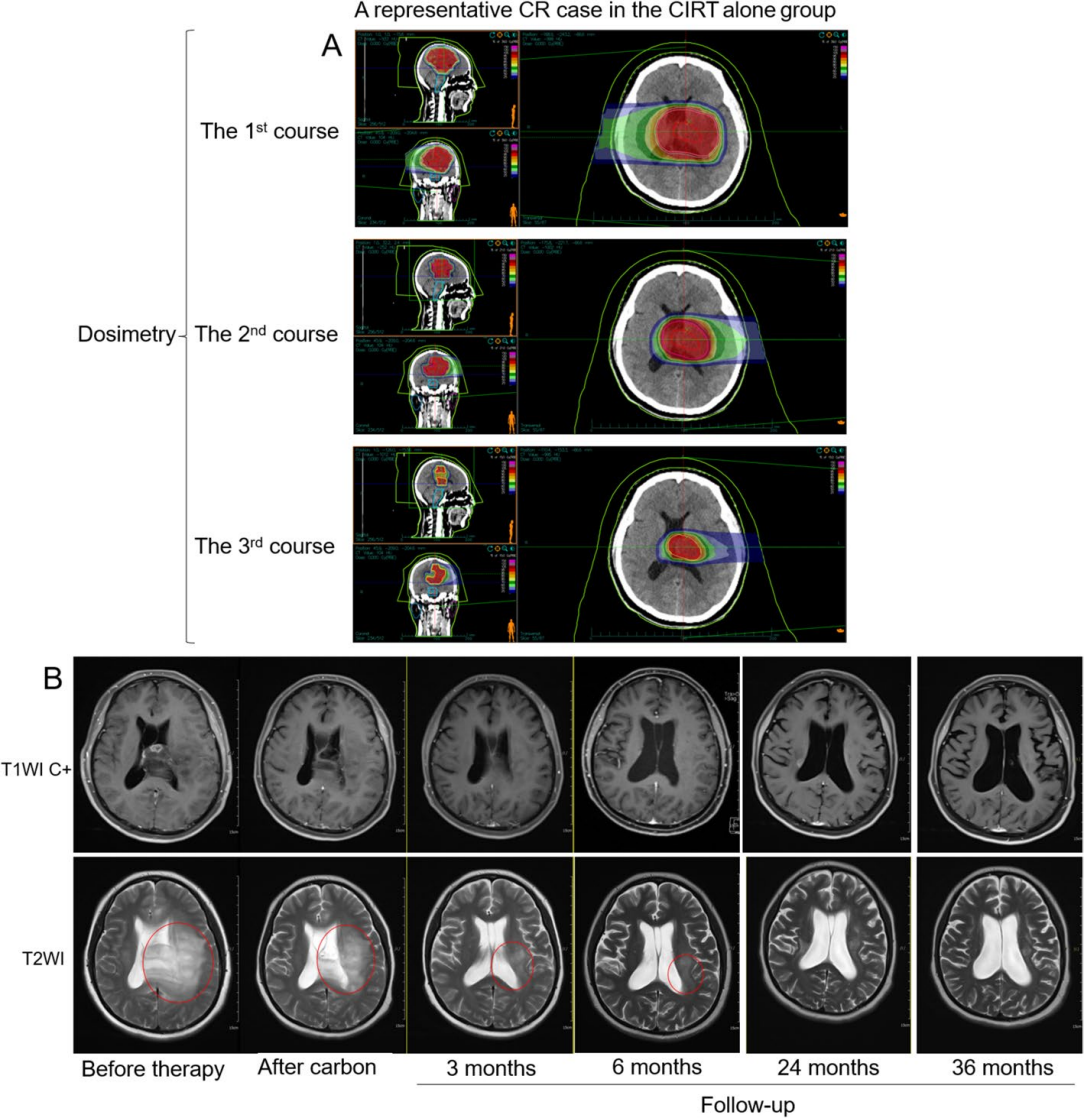
Representative images of a CR case in the CIRT alone group. The dosimetry (**A**) and MRI (**B**) images of a CR case in the CIRT alone group were shown. In a follow-up series at the indicated time points, tumor residual is visualized by MRI and highlighted by red frames

**Fig. 6:**
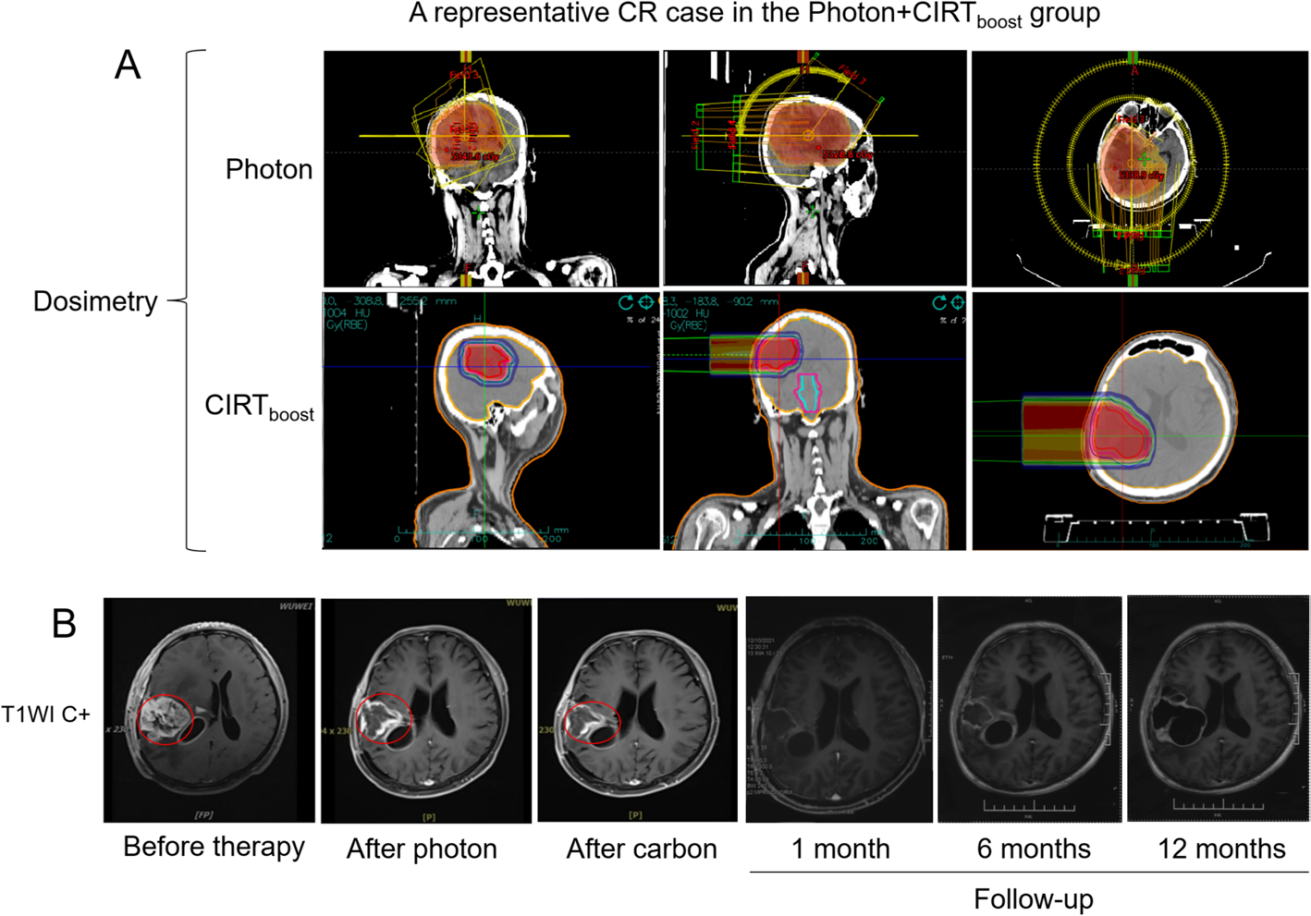
Representative images of a CR case in the Photon + CIRT_boost_ group. The dosimetry (**A**) and MRI (**B**) images of a CR case in the Photon + CIRT_boost_ group were shown. In a follow-up series at the indicated time points, tumor residual is visualized by MRI and highlighted by red frames

To explore the clinical characteristics that might influence survival in patients who received CIRT alone or Photon + CIRT_boost_, we performed subgroup PFS and OS analysis. For CIRT alone therapy, female patients and patients with IDH1/2 mutations had significantly better PFS (Fig. 3A and C) and OS (Fig. 4A and C) compared to their respective counterparts. No significant difference was observed by WHO tumor grade or KPS score separation (Figs. 3B and D and 4B and D). For Photon + CIRT_boost_ therapy, no significant difference in PFS or OS was observed in all subgroup analyses (Figs. 3E-H and 4E-H). The typical dosimetry and MRI images of two representative CR cases in CIRT alone (Fig. 5A-B) and Photon + CIRT_boost_ (Fig. 6A, B) were provided. For CIRT alone, three layered doses were designed, including GTV: 60.0 Gy (RBE)/20 Fx, V1: 30.0 Gy (RBE)/10 Fx, V2: 45.0 Gy (RBE)/15 Fx. Sequential boosting was performed using a three-course plan, gradually decreasing the target area (Fig. 5A).

### Acute and late toxicities

Only grade 1 acute toxicities were observed in CIRT alone and Photon + CIRT_boost_ groups (Table 4). CIRT alone group had a significantly lower ratio of acute toxicities compared to Photon + CIRT_boost_ (3/18 vs. 9/16,* p* = 0.03) (Table 4). Similar rates of late toxicities were observed between the two groups (5/18 vs. 2/16,* p* = 0.40) (Table 4). However, all late toxicities of Photon + CIRT_boost_ were grade 1. 4/5 were grade 2 in the CIRT alone group, including one case of cerebral necrosis (Table 4).

**Table 4:** Comparison of acute and late toxicities between CIRT alone and Photon + CIRT_boost_ groups

Toxicities type	Group	No of cases	Fisher's exact test	Toxicities score		
				**G1**	**G2**	**G3**
**Acute toxicities**	CIRT alone (*N* = 18)	3	*p* = 0.030	Cerebral edema, *n* = 3		
	Photon + CIRT_boost_ (*n* = 16)	9		Cerebral edema, *n* = 4 Alopecia, *n* = 2 Adiodermatitis, *n* = 2 Headache, *n* = 1		
**Late toxicities**	CIRT alone (*N* = 18)	5	*p* = 0.40	Cerebral edema, *n* = 1	Cerebral edema, *n* = 3 Cerebral necrosis, *n* = 1	
	Photon + CIRT_boost_ (*n* = 16)	2		Cerebral edema, *n* = 2		

## Discussion

Over the past 10 years, there has been general agreement about how to manage gliomas. For newly diagnosed HGGs, the standard treatment approach is maximally safe surgery, concurrent chemotherapy with temozolomide (75 mg/m^2^ daily for 42 days) plus radiotherapy, additional chemotherapy with temozolomide (150–200 mg/m^2^ for 5 days every 28 days) for 6–12 cycles, in combination with tumor treatment fields (TTF) in some countries [12, 15]. Patients enrolled in clinical trials testing new systemic therapies and those treated in regular clinical practice, typically receive 60 Gy of radiation delivered in 30 fractions [27]. This regimen has become the standard after multiple prior failed attempts at dose escalation, including hyperfractionation [2], stereotactic radiosurgery [22, 26], and brachytherapy boosts [16]. Despite promising results from phase I studies and modern dose escalation approaches [25], the NRG Oncology BN001 phase II study (NCT02179086) reconfirmed the lack of benefit from escalating the photon radiation dose to 75 Gy in 30 fractions, even with concurrent radiosensitizing chemotherapy [10]. Median OS was still 18.7 months, without significant improvement compared to standard-dose (60 Gy) [10].

In addition to clinical trials evaluating the dose and fractionation of photon radiation, recent research has also concentrated on the dosimetric and physical properties of particle therapies to improve tumor control. However, a randomized, prospective phase II trial found no difference in the onset of cognitive decline between proton therapy and modern photon techniques [5]. A secondary analysis also found no differences in PFS or response assessment (Al [1]). Carbon-ion beam-based strategies were also explored in clinical settings, either alone or as a boost after initial proton or photon therapy. A recent study reported the first use of particle therapy plus concurrent temozolomide to treat high-grade gliomas. They observed 18-month OS and PFS rates of 72.8% and 59.8%, respectively, with CIRT_boost_ therapy and temozolomide [13]. In this study, we observed that in grade 4 HGG in received Photon + CIRT_boost_ therapy, the 12- and 18-month PFS rates were 66.7% (95%CI, 27.2%-88.1%) and 50.0% (95%CI, 13.4%-78.7%), while the 12- and 18-month OS rates were 87.5% (95%CI, 38.7%-98.1%) and 72.9% (95%CI, 27.6%-92.5%). These findings are consistent with data from Kong et al.. The median OS was 19 months and the 24-month OS rate drastically dropped to 48.6% (95%CI, 7.7%-81.6%). CIRT alone had no statistically inferior effect in terms of PFS and OS compared to Photon + CIRT_boost_ therapy.

Our subgroup analysis found that when CIRT was provided alone, the PFS and OS differences in patients stratified by gender and IDH1/2 mutation status were statistically significant. Thu, these characteristics should be carefully considered if CIRT was provided alone. However, these subgroup data should be carefully interpreted since the sample size in each subgroup is relatively small. In addition, for the difference in grade separation, only 2 or 3 grade 3 patients were included in one group. We could not make reliable conclusions based on such a small sample size. In patients with Photon + CIRT_boost_ therapy, these characteristics might not affect survival outcomes.

In terms of acute and late toxicity, only grade 1 toxicities were observed in Photon + CIRT_boost_, although it had a significantly higher ratio of acute toxicities than CIRT alone. Grade 3 toxicities were not observed in both therapeutic strategies.

This study also has several limitations. Firstly, the relatively small number of patients reviewed might hamper the statistical power. Secondly, this study is a retrospective analysis. Potential selection bias was inevitable. Thirdly, over 50% of patients in this study had no MGMT promoter methylation status information, making subgroup analysis impossible. However, the insights gained from this study lay the groundwork for subsequent prospective randomized trials. By highlighting the safety profile and potential efficacy of CIRT, either alone or as a Photon + CIRTboost, our research identifies key questions and considerations for future investigations. Thirdly, the recent surge in the popularity of such non-linear models highlights their potential advantages over traditional Cox proportional hazards regression in oncology research. For example, ensemble learning methods might capture complex interactions and nonlinear relationships in the data, potentially leading to improved predictive performance compared to standard parametric or semiparametric survival models [20]. Bayesian additive regression trees (BART) and soft BART have demonstrated promising results in various survival analysis settings, including clustered and interval-censored data [3, 17]. Additionally, the incorporation of grouping information, as described in the work by Du and Linero [8], can be particularly relevant for our study, where we stratified the analysis by patient characteristics like gender and IDH mutation status. Such group-based ensemble approaches may provide additional insights into the heterogeneous treatment effects observed in our cohort in the future.

## Conclusion

CIRT alone and Photon + CIRT_boost_ with concurrent temozolomide are safe, without significant differences in PFS and OS in HGG patients. In grade 4 HGG received Photon + CIRT_boost,_ the 12- and 18-month PFS rates were 66.7% (95%CI, 27.2%-88.1%) and 50.0% (95%CI, 13.4%-78.7%), while the 12- and 18-month OS rates were 87.5% (95%CI, 38.7%-98.1%) and 72.9% (95%CI, 27.6%-92.5%), which is comparable to the dose-intensification group in the NRG Oncology BN001 phase II study. It is meaningful to explore whether dose escalation of CIRT_boost_ might improve survival outcomes of HGG patients in future randomized trials.

### Supplementary Information

Additional file 1: Supplementary Table 1. Univariate analysis for PFS in all HGG cases. Supplementary Table 2. Univariate analysis for OS in all HGG cases. Supplementary Table 3. Univariate analysis for PFS in grade 4 HGG cases. Supplementary Table 4. Univariate analysis for OS in grade 4 HGG cases.

## Data Availability

The datasets used and analyzed during the current study are available from the corresponding author on reasonable request.
