# Supplementary material for: Carbon-ion radiotherapy alone vs. standard dose photon radiation with carbon-ion radiotherapy boost for high-grade gliomas: a retrospective study
Source: BMC Cancer. 2024 Jul 13;24:837. doi: 10.1186/s12885-024-12606-x (PMC11245814; doi:10.1186/s12885-024-12606-x)
Supplement: Supplementary file 1 — Additional file 1: Supplementary Table 1. Univariate analysis for PFS in all HGG cases. Supplementary Table 2. Univariate analysis for OS in all HGG cases. Supplementary Table 3. Univariate analysis for PFS in grade 4 HGG cases. Supplementary Table 4. Univariate analysis for OS in grade 4 HGG cases. [file 12885_2024_12606_MOESM1_ESM.docx]

**Supplementary Table 1. Univariate analysis for PFS in all HGG cases**

| Characteristics | Total (N) | Univariate analysis | |  |
| --- | --- | --- | --- | --- |
|  |  | Hazard ratio (95% CI) | *p* value |  |
| Treatment | 34 |  |  |  |
| P+C | 16 | Reference |  |  |
| C | 18 | 1.678 (0.589 - 4.780) | 0.332 |  |

P+C: Photon+CIRT_boost_; C: CIRT alone

**Supplementary Table 2. Univariate analysis for OS in all HGG cases**

| Characteristics | Total (N) | Univariate analysis | |  |
| --- | --- | --- | --- | --- |
|  |  | Hazard ratio (95% CI) | *p* value |  |
| Treatment | 34 |  |  |  |
| P+C | 16 | Reference |  |  |
| C | 18 | 0.926 (0.278 - 3.083) | 0.900 |  |

P+C: Photon+CIRT_boost_; C: CIRT alone

**Supplementary Table 3. Univariate analysis for PFS in grade 4 HGG cases**

| Characteristics | Total (N) | Univariate analysis | |  |
| --- | --- | --- | --- | --- |
|  |  | Hazard ratio (95% CI) | *p* value |  |
| Treatment | 28 |  |  |  |
| P+C | 14 | Reference |  |  |
| C | 14 | 1.546 (0.527 - 4.535) | 0.427 |  |

P+C: Photon+CIRT_boost_; C: CIRT alone

**Supplementary Table 4. Univariate analysis for OS in grade 4 HGG cases**

| Characteristics | Total (N) | Univariate analysis | |  |
| --- | --- | --- | --- | --- |
|  |  | Hazard ratio (95% CI) | *p* value |  |
| Treatment | 28 |  |  |  |
| P+C | 14 | Reference |  |  |
| C | 14 | 1.152 (0.347 - 3.822) | 0.818 |  |

P+C: Photon+CIRT_boost_; C: CIRT alone
